# View-flipping effect reduction and reconstruction visualization enhancement for EPISM based holographic stereogram with optimized hogel size

**DOI:** 10.1038/s41598-020-70256-y

**Published:** 2020-08-10

**Authors:** Xingpeng Yan, Teng Zhang, Chenqing Wang, Yunpeng Liu, Ziqiang Wang, Xi Wang, Zhe Zhang, Min Lin, Xiaoyu Jiang

**Affiliations:** 1 Department of Information Communication, Academy of Army Armored Forces, Beijing, 100072 China; 2 Department of Basic Military and Political Education, Academy of Army Armored Forces, Beijing, 100072 China; 3 Department of Basic Education, Academy of Army Armored Forces, Beijing, 100072 China

**Keywords:** Imaging and sensing, Displays

## Abstract

To reduce the view-flipping effect and enhance the viewing resolution, the modulation characteristics of the hogel based holographic stereogram is constructed and validated. The performance of the view-flipping effect is analyzed, and the results indicate that decreasing the size of hogel is beneficial to the reduction of the view flipping, however, which will result in significant diffraction effect which can degrade the reconstruction quality. Furthermore, a diffraction-limited imaging model of the hogel based holographic stereogram is established, where both the limited aperture of the hogel and the defocused aberration of the object point are introduced, and the effective resolvable size of the reconstructed image point is simulated. The theory shows that there is an optimal hogel size existed for the certain depth of scene. Both the numerical and optical experiments are implemented, and the results are well agreed with the theoretical prediction, which demonstrates that the view-flipping reduction and reconstruction visualization enhancement for EPISM based holographic stereogram can be achieved when the proper size of hogel is utilized.

## Introduction

Three dimensional (3D) display has attracted an intense research interest in recent years^1–10^, and it also has potential applications in variable fields, such as industrial, medical, military, artistic, and so on^11^. Holography is a promising approach to get realistic 3D reconstruction^12,13^. Traditional hologram is capable of reproducing extremely high-quality 3D images by wavefront reconstruction, which can be achieved by the way of interference between the object light and the reference light. Similarly, the computer-generated hologram (CGH) also records the wavefront information by numerical calculation and electron beam lithography/laser direct writing^14–16^. CGH is ideally suited to transmission holography and has no ability to print reflection hologram. Some other holographic technologies have also been developed due to the progress on novel materials or devices^17–21^. Compared to the traditional hologram as well as the CGH, holographic stereogram can reconstructed real or virtual objects by printing reflection hologram with high-fidelity, distortion-free and full-color multiple reconstructed 3D images^22–24^. A series of perspective images is obtained recoded to expose into small holographic elements, which are called hogels. The holographic stereogram has become the focus of research^8,25–29^, and are also widely applied in many fields^30^.

As early as 1967, Pole photographed the 3D scene illuminated with ordinary white light by a fly’s eye lens to generate a "holocoder", and then converted the "holocoder" into a Fresnel hologram^22^. According to this approach, the virtual stereo images of the 3D object can be reconstructed. In 1969, the slit was added into holographic stereogram printing system creatively by DeBitetto^23^ to improve the poor resolution of holographic stereogram. In 1970, King fabricated a holographic stereogram by DeBitetto’s process and printed its Fresnel hologram furtherly to obtain a white light-viewable image plane hologram^24^. Considering the cost of time, the final holographic stereogram usually contained only horizontal parallax. In 1990s, most large reflection holographic stereograms had therefore started to be recorded using the DeBitetto/King model^23,31,32^. In 1991, the ultragram was described by Halle et al.^33^, in which the relationship between capture and recording geometries was relaxed and the viewer can still get 3D stereoscopy at arbitrary plane. In 1992, Yamaguchi proposed a new holographic stereogram printer^34^, which can make accurate distortionless hard copies of 3 D object with both horizontal and vertical parallaxes. In the next year, Yamaguchi analyzed the wavefront reconstruction of holographic stereogram and proposed phase added stereogram (PAS)^35^, which required less computation load than actual CGH and presented higher reconstruction quality. In the late 1990s, Klug et al. presented an apparatus and method for printing one-step, full-color, full-parallax holographic stereograms^36^. Bjelkhagen and Brotherton-Ratclife extensively reviewed direct-write digital holography (DWDH)^11,37,38^, which can be traced back to as early as 2002. In 2017, a new method of single-step full parallax holographic stereogram printing is proposed by our group, which based on effective perspective images’ segmentation and mosaicking (EPISM)^39^. The holographic stereogram, printed based on EPISM, had good reconstructed images and low time cost.

The reconstruction quality evaluation of 3D imaging system has always been the difficulty of the research, so does holographic stereogram. In 1991, Halle et al. designed a test pattern to analyze distortion of holographic stereogram, which consists of three hollow squares all having the same height and width but located at different depths, and proposed the infinite viewpoint camera approach to compensate the distortion^33^. Meanwhile, he found that the discrete nature of the stereogram is the underlying cause of view-flipping and proposed that smaller slits and more capturing images can reduce the problem of view-flipping^40^. As early as 1994, Pierre St Hilaire^41^ investigated the modulation transfer function (MTF) characteristics of image-plane holographic stereograms and determined the optimum stereogram parameters. The related research by theory analysis can lead to significant computational economies by appropriate choice of number of perspectives, image resolution, and scene depth. In 2006, Takaki constructed a prototype display, in which the discrepancy between the convergence function and the accommodation function was eliminated so that the display does not induce view-flipping effect^42^. In 2006, L. E. Helseth obtained an average sampled modulation transfer function describing holographic stereograms and theoretical analysis showed that an increasing slit size reduced the optical resolution of the system and Gaussian slit may improve the optical transfer function (OTF) for small spatial frequencies^43^. In 2018, Jani Makinen et al*.* provided analysis of the relation between the hologram sampling properties and the perceived accommodation response, and further demonstrated that the accommodation response can be enhanced at the expense of loss in perceived spatial resolution^44^. In 2018, our group modelled the OTF of the full parallax EPISM printing system from the aspect of frequency domain, hogel size and the sampling interval of original perspective images are both optimized to improve the imaging quality^45^. In the same year, our group further discussed the view-flipping effect in the spatial domain and evaluate the reconstructed quality by optical transfer function of the EPISM-based holographic stereogram^46^, and showed that the view-flipping effect can be improved significantly with appropriate hogel sizes. The similar issue of motion parallax performance has also been investigated in some other optical filed display systems especially in integral imaging^47,48^. In 2019, Wang et al. proposed the concept of resolution priority holographic stereogram based on integral imaging for the first time, and a multi-plane technique as well as multi-exposure technique was used to print the hogel to enhance the depth range^49^. However, the imaging theory of EPISM is different from that of the integral imaging, which is mainly reflected in the data pickup strategy, optical information coding algorithm and the reconstruction principle, and this will give rise to different countermeasure to enlarge the display depth of field and smooth the motion parallax. Generally speaking, some previous researches indicated that the smaller the size of slit or hogel is, the better reduction of the view-flipping effect is, and the view flipping can be eliminated under very small size of slit or hogel, which is essential to the motion parallax of the holographic stereogram. The other researches applied the OTF or MTF to describe the transformation performance of the spatial frequency, but it is focused on the performance of each single reconstructed 3D image. For holographic stereogram, the reconstruction resolution of the reconstructed 3D image when observed at the certain angular position, and the continuous motion parallax when perceived from different viewing angles, are both play an equally and extremely important role on the visual experience. Therefore, it is significant to simultaneously reduce the view-flipping effect and enhance the reconstruction visualization when a hogel based holographic stereogram is designed and fabricated.

In this work, the view-flipping effect is investigated in detail, where the flipping distance and the flipping angle are introduced and formulated to evaluate the scale of the flipping effect. The analysis shows that the view-flipping effect can be eliminated as much as possible by reducing the size of hogel. Considering the diffraction effect caused by small size hogel, a diffraction-limited imaging model of the hogel based holographic stereogram is further established, and the point spread function is employed to evaluate the resolution characteristics of the imaging system. Both view-flipping effect and resolution characteristics are considered as factors to improve the imaging performance. According to the analysis, a compromise between the view-flipping effect and the viewing resolution can be realized, and a better reconstruction quality can be obtained when an optimized hogel size is utilized. The experimental results verify the theoretical predictions, and demonstrate that the view-flipping reduction and reconstruction visualization enhancement can be achieved in holographic stereogram by utilizing optimized hogel size.

## View-flipping effect in holographic stereogram

EPISM method is a holographic stereogram printing method proposed firstly by our groups, and generate direct H_2_—pop-out images—which are the most fascinating for the public^39^. EPISM method can be divided into three steps: The first step is to sample the virtual or real 3D scene from different perspectives by camera array, and the sampling range is bounded by the perceptual frustrum of the 3D scene. In the sampling, the number of capturing images is determined by camera interval and array size, and the resolution of capturing images should be set to match the resolution of the desired synthetic parallax images. The second step is a crucial step. The sampled image is segmented and mosaicked to generate synthetic parallax images, so that the reconstructed 3D scene has correct parallax perception and depth cues. The quality of the synthesized parallax images is mostly determined by the number of capturing images. The more capturing images, the better synthetic parallax images, however, which also rises more complexity. The detailed principle as well as the algorithm of segmentation and mosaicking is described in our previous work. Finally, synthetic parallax images are loaded on the spatial light modulator (SLM) one by one until the end of the printing process.

The view-flipping effect is an inherent shortcoming for stereogram, including the holographic stereogram. In stereogram, essentially, a limited number of perspective images are coded to appropriate the continuous optical field of 3D scene, and if the constructed parallax relationship between these perspective images is identical to the actual viewing effect of the 3D scene, a 3D imaging can be realized due to the binocular disparity. However, restricted by the discrete and limited number of perspective images, a discontinuity of viewing parallax is resulted in when the reconstruction is perceived with continuously angular moving. This discontinuous parallax can lead to the ocular fatigue and decrease the visual effect of the constructed 3D images. Therefore, in order to improve the reconstruction quality and the visual experience, the view-flipping effect should be reduced or eliminated.

**Figure 1:**
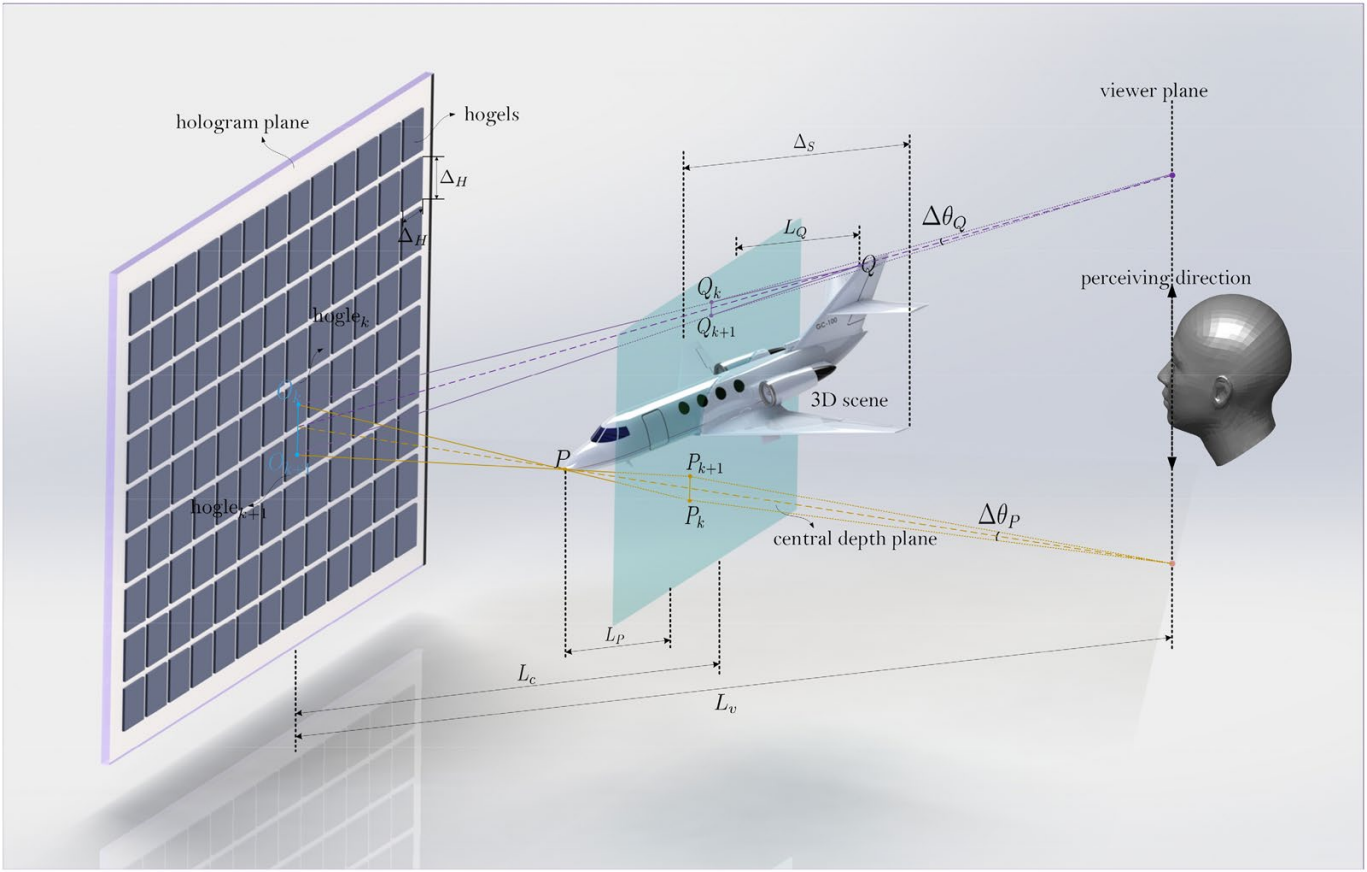
The diagram of the recording/reconstruction of hogel based holographic stereogram.

The recording/reconstruction configuration of holographic stereogram is shown in Fig. 1. The hogel size is denoted as $\Delta_{H}$, and the central depth plane (CDP) of the 3D scene, which is usually defined as the plane of geometric central plane of the 3D scene along the depth direction, is placed $L_{c}$ away from the hologram plane, while the depth of the 3D scene is supposed as $\Delta_{S}$. The hologram is watched by the viewer at the distance of $L_{v}$, and the CDP is assumed to place between the hologram plane and the viewer, i.e., $L_{v} > L_{c}$. In order to show the details better, the size of hogel in Fig. 1 is depicted much larger than the actual one, and the head image is relatively smaller. Consider an object point $P$ located on the nearer side (as seen in Fig. 1, on the left side of CDP) of the 3D scene, and there are two hogels, ${\text{hogel}}_{k}$ and ${\text{hogel}}_{k + 1}$, located on the hologram plane, with their center points $O_{k}$ and $O_{k + 1}$ respectively. The line $PO_{k}$ and $PO_{k + 1}$ intersect the central depth plane at point $P_{k}$ and $P_{k + 1}$. Thus, when perceive the reconstructed 3D scene, the points $P_{k}$ and $P_{k + 1}$ are the corresponded stereo disparities of ${\text{hogel}}_{k}$ and ${\text{hogel}}_{k + 1}$. In other words, during the exposure process, the point $P_{k}$ is coded to a synthetical perspective image and exposed to ${\text{hogel}}_{k}$, while $P_{k + 1}$ is exposed to ${\text{hogel}}_{k + 1}$. When the hologram is optically reconstructed, the parallax point $P_{k}$ of object point $P$ will be perceived when the viewer’s eye pupil is placed on the line $PO_{k}$. If the viewer’s eye pupil moves to line $PO_{k + 1}$, another parallax point ${\text{hogel}}_{k + 1}$ then will be perceived. If the included angle $\theta_{P}$ is less than the minimum angular resolvable limit of human eye which is often chosen as about $\theta_{{\min}} = 0.3\;{\text{mrad}}$^10^, the continuous motion parallax can be achieved without the presence of view-flipping effect. In 3D display system, $\theta_{{\min}} = 0.3\;{\text{mrad}}$ is such an exacting criteria that the minimum angular resolvable limit of human eye can be relaxed appropriately to some extent, here $\theta_{{\min}} = 1.5 \times 0.3\;{\text{mrad}}$ is employed. On the contrary, if $\theta_{P} > \theta_{{\min}}$, a distinctly perceptible parallax difference can be observed where parallax point $P_{k}$ jumps discretely to $P_{k + 1}$, and thus the view-flipping effect occurs. The flipping distance $\Delta P_{k,k + 1} = \overline{{P_{k} P_{k + 1} }}$ can be represented as follows,

1 $$\Delta P_{k,k + 1} = \frac{{\Delta_{H} \times L_{P} }}{{L_{c} - L_{P} }}$$

where $L_{P}$ is the distance between the object point $P$ and the central depth plane.

Similarly, for an object point $Q$ located on the further side (see Fig. 1, on the right side of CDP) of the 3D scene, the corresponding flipping distance $\Delta Q_{k,k + 1} = \overline{{Q_{k} Q_{k + 1} }}$ can be formulated as

2 $$\Delta Q_{k,k + 1} = \frac{{\Delta_{H} \times L_{Q} }}{{L_{c} + L_{Q} }}$$

where $L_{Q}$ is the distance between the object point $Q$ and the central depth plane. For actual holographic stereogram, the size of the hogel is often small enough that the span angle between two adjacent parallax points for $P$ and $Q$ can be expressed as

3 $$\Delta \theta_{P} \approx \frac{{P_{k,k + 1} }}{{L_{v} - L_{c} }} = \frac{{\Delta_{H} \times L_{P} }}{{(L_{v} - L_{c} )(L_{c} - L_{P} )}}$$

and

4 $$\Delta \theta_{Q} \approx \frac{{Q_{k,k + 1} }}{{L_{v} - L_{c} }} = \frac{{\Delta_{H} \times L_{Q} }}{{(L_{v} - L_{c} )(L_{c} + L_{Q} )}}$$

where $\Delta \theta_{P,Q}$ can be defined as the angular resolution.

From Eqs. (3) and (4), it can be seen that the view-flipping effect is influenced by hogel size $\Delta_{H}$ the location of the central depth plane $L_{c}$, the object point’s location $L_{P} /L_{Q}$, and the viewer’s watching distance $L_{v}$. The smaller the $\Delta_{H}$ is, the fewer the $\Delta \theta_{P,Q}$ acts, which means that with smaller size of hogel, the view-flipping effect can be reduced. If the location of the central depth plane is putting further away from the hologram plane, i.e., with larger $L_{c}$, the smaller $\Delta \theta_{P,Q}$ can be obtained. Meanwhile, if we observe the reconstructed 3D scene further away from the hologram, the view-flipping effect would also be alleviated. For object points either on the further side or on the nearer side, the more severe view-flipping can be induced when the object point locates further away from the central depth plane, and the minimum view-flipping effect can be obtained when the object point is on the central depth plane. It should be pointed out that if the object points on the nearer side is located on the hologram plane, i.e., $L_{c} - L_{P} = 0$, the view-flipping effect may trend to infinity. In other words, we cannot get an image hologram from a one-step holographic stereogram. If the object point is located behind the hologram ($L_{c} < L_{P}$), an inverted view-flipping effect can be perceived. The above conclusion is always true for all object points on the 3D scene.

Furthermore, combine Eqs. (3) and (4), we have

5 $$\frac{{\Delta \theta_{P} }}{{\Delta \theta_{Q} }} = \frac{{L_{c} + L_{Q} }}{{L_{c} - L_{P} }} > 1,\quad {\text{when}}\quad L_{P} = L_{Q}$$

**Figure 2:**
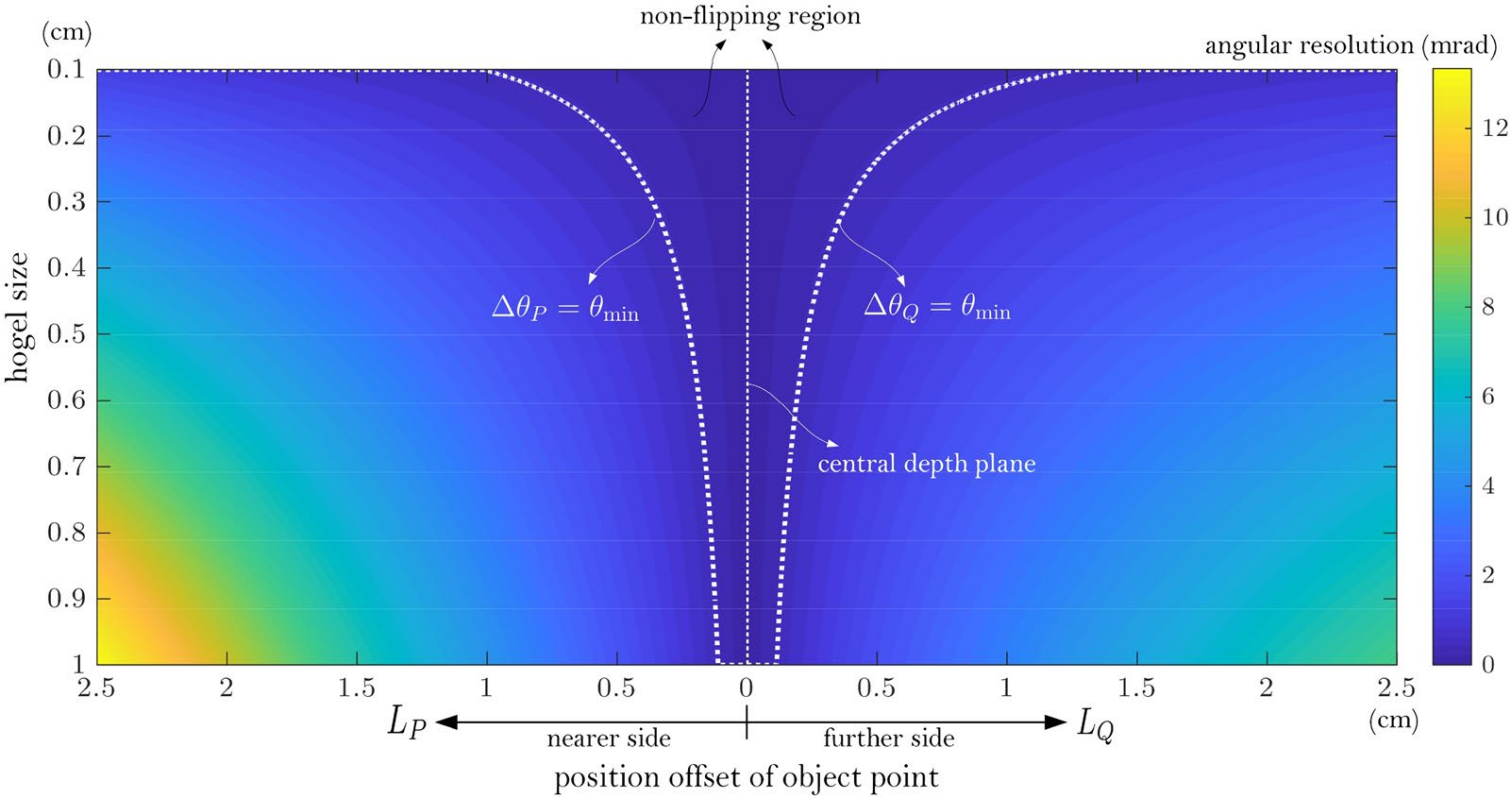
Numerical example of view-flipping effect in the terms of angular resolution $\Delta \theta_{P,Q}$. $L_{v} = 35$ cm, $L_{c} = 10$ cm, $\theta_{{\min}} = 0.45\;{\text{mrad}}$.

It shows that all the object points on the nearer side exhibit a more severe view-flipping effect than that on the further side when they have the same distance from the CDP, i.e., $L_{P} = L_{Q}$. The $\Delta \theta_{P,Q}$ is calculated and is illustrated in Fig. 2 to show the conclusion more intuitively, where the non-flipping region is also illustrated.

Here the viewer’s watching distance is set as $L_{v} = 35$ cm since the comfortable viewer’s watching distance can often be supposed within the range of 25 cm ~ 50 cm. The position of the central depth plane is chosen as $L_{c} = 10$ cm. A larger $L_{c}$ means that the reconstructed 3D scene is put further away from the hologram plane which would reduce the field of view of the reconstruction, thus $L_{c}$ should not be too large. On the other hand, $L_{c}$ should not be too small yet because a smaller $L_{c}$ can lead to the rising of flipping distance according to Eqs. (1) and (2). Under the fixed viewer’s watching distance $L_{v}$ and CDP position $L_{c}$, the view-flipping effect can be eliminated when either the object point’s position offset $L_{P,Q}$ or the hogel size $\Delta_{H}$ should be small enough. However, once the 3D scene is selected, the characters of its object points’ position offset $L_{P,Q}$ is also determined. It is obvious that the only way to reduce or eliminate the view-flipping effect is to diminish the hogel size $\Delta_{H}$. The decrease of hogel size can lead to a significant increase of time cost for the holographic stereogram fabrication. For example, the fabrication time will be increased by four times if the hogel size is diminished by half. More importantly, the hogel size $\Delta_{H}$ also plays an important role on the reconstructed display quality of the holographic stereogram, especially the diffraction effect of the hogel aperture becomes serious, which is analyzed in the following section.

## Modeling of diffraction-limited imaging of hogel based holographic stereogram

The previous researches on the resolution performance of holographic stereogram are almost based on the principle of geometrical optics, where the diffraction effect of the hogel window is neglected, and the rays are assumed to propagate along straight line during both the recording and reconstruction process. This assumption is valuable when the size of the hogel is large enough. When the hogel’s window size decreases, the diffraction effect arises and should be taken into account. From the analysis mentioned above, the non-flipping is always achieved when the hogel size $\Delta_{H}$ is small enough. Thus, a more quantitative analysis for the selection of hogel size $\Delta_{H}$ becomes imperative and should be modeled.

The recording and reconstruction process of the holographic stereogram is a typical optical imaging system. Due to the limited size of hogel, the narrow-band light source used to fabricate and reconstruct the hologram, as well as the definite depth of the reconstructed 3D scene, this system can be regarded as a diffraction-limited imaging system. The characteristics of the reconstructed 3D image is dominated by the diffraction effect as well as the aberration. The imaging of the holographic stereogram is similar to that of the lens, which merely separates the 3D image to its original 3D object in both time domain and spatial domain. The 3D information of the original object is stored into the holographic medium as interference patterns by the interference between the object beam and the reference beam, and the reconstruction of the 3D scene is a process that restored the recorded 3D information by diffraction.

**Figure 3:**
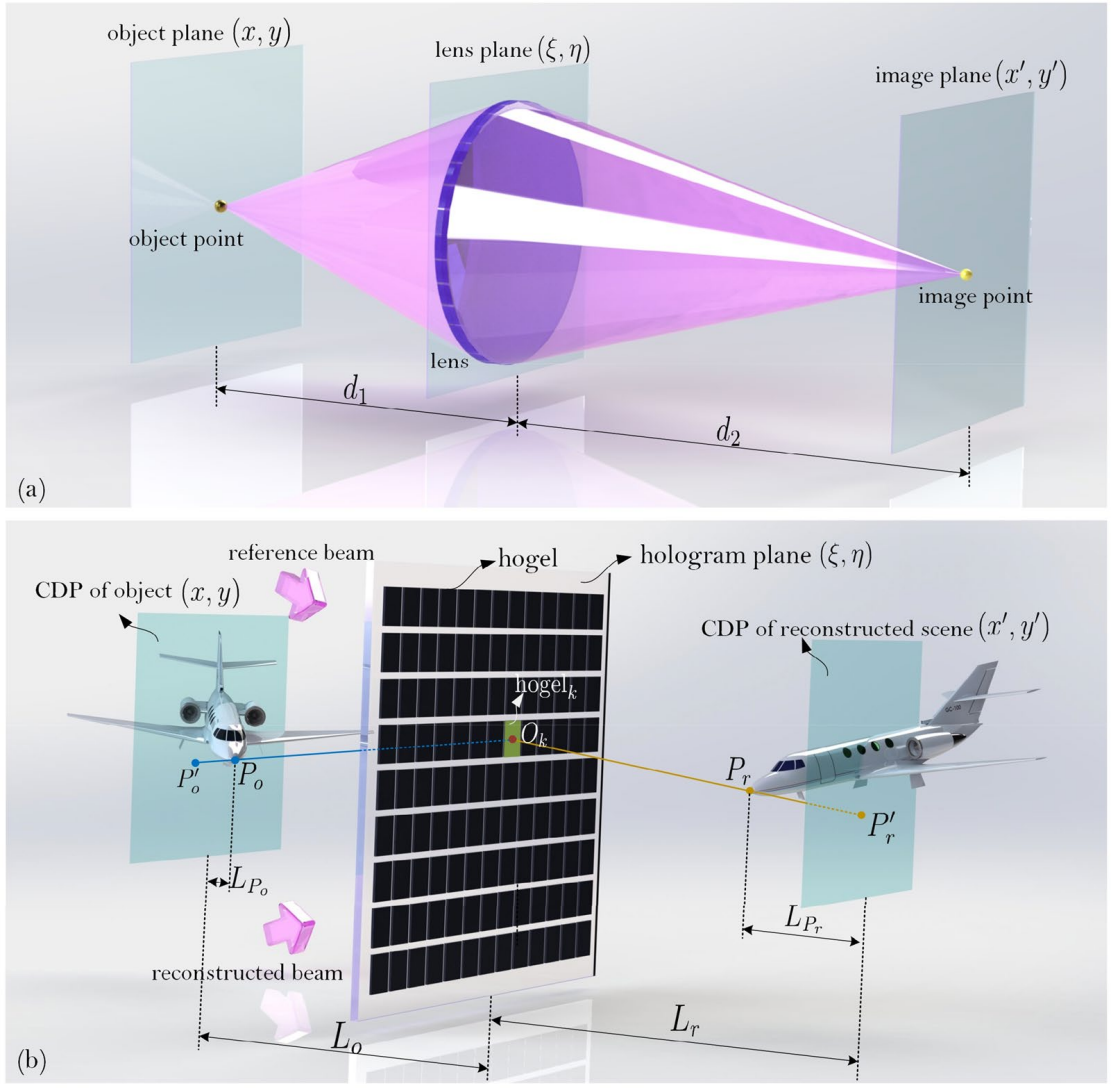
(**a**) The imaging model of the lens and (**b**) the equivalent imaging model for the recording and reconstruction of hogel based holographic stereogram.

Under the assumption of monochromatic illumination with light wavelength of $\lambda$, the imaging system employed a thin lens aberration-free lens with the focal length of $f$ is shown in Fig. 3a. The complex amplitude of the object point $P$ can be consider as an ideal point source, then

6 $$U(x,y) = \delta (x,y)$$

and the complex amplitude transmittance function of the lens is

7 $$t(\xi ,\eta ) = T(\xi ,\eta )\exp \left[ { - {\text{j}}\frac{k}{2f}(\xi^{2} + \eta^{2} )} \right]$$

where the lens’ aperture function is

8 $$T(\xi ,\eta ) = \left\{ {\begin{array}{*{20}c} {1,} & {\sqrt {\xi^{2} + \eta^{2} } \le r} \\ {0,} & {\sqrt {\xi^{2} + \eta^{2} } > r} \\ \end{array} } \right.$$

and $k = 2\pi /\lambda$ is the wave number, ${\text{j}} = \sqrt { - 1}$, $r$ is the radius of the imaging lens.

After imaging by the lens, the complex amplitude of image point $P^{\prime}$ is^50^

9 $$\begin{gathered} U^{\prime}(x^{\prime},y^{\prime}) = \frac{1}{{\lambda^{2} d_{1} d_{2} }}\int {\int_{ - \infty }^{ + \infty } {T(\xi ,\eta )\exp \left[ { - {\text{j}}\frac{k}{{d_{2} }}(\xi x^{\prime} + \eta y^{\prime})} \right]{\text{d}}\xi {\text{d}}\eta } } \hfill \\ \hfill \\ \end{gathered}$$

where $d_{1}$ is the distance between the object plane and the lens plane, $d_{2}$ is the distance between the lens plane and the image plane, and they satisfy $d_{1}^{ - 1} + d_{2}^{ - 1} = f^{ - 1}$.

It is obvious that if the radius of the imaging lens trends to infinity, i.e., $r \to \infty$, the image point is an ideal point. For a practical lens with finite radius $r$, the diffraction effect of the limited lens’ aperture must be taken into account, and the image point is not an ideal point any more, but a blurring cloud-like diffraction spot. Thus, the size of the lens’ aperture plays an important role on the imaging performance. If $d_{1} = 2f$, we have $d_{1} = d_{2}$, and then the system can project the object to its inverted real image with the same size, i.e., its magnification is $M = - d_{2} /d_{1} = - 1$. Similarly, when the holographic stereogram is illuminated by the conjugate beam of the original reference light, an up-right 3D image with the same size of the original object can be reconstructed. Thus, similar to Eq. (7), we can derive the transformation function of the complex amplitude for holographic stereogram. An equivalent imaging model for the recording and reconstruction of the EPISM is shown in Fig. 3b. The 3D scene $U(x,y,z)$ with its central depth plane is imaged by the holographic stereogram, and an up-right conjugate 3D image $U^{\prime}(x,y,z)$ with the same size is reconstructed.

According to the principle of EPISM^39^, the nature of the above equivalent imaging system is that, the effectively synthetic perspective image on the CDP of object is imaged holographically to the reconstructed CDP of image with magnification $M = 1$. As shown in Fig. 3b, the process of the recording of the EPISM based holographic stereogram and its reconstruction are interpreted as an imaging system, where the reconstructed scene with real pseudoscopic images is exemplified for simplicity. The effectively synthetic perspective images are placed at the location of the CDP of the original 3D scene and are then exposed to the hogels located on the hologram plane, and are further reconstructed onto the CDP of the reconstructed 3D scene. Thus, central depth plane of the original 3D scene and that of the reconstructed 3D scene are conjugated, in other words, they are mirror images referred to the hologram plane. For the specified ${\text{hogel}}_{k}$ with its center point $O_{k}$, an object point $P_{o}$ can be mapped to the pixel point $P^{\prime}_{o}$ on the effectively synthetic perspective image corresponding to ${\text{hogel}}_{k}$. After recording and reconstruction, it was reconstructed as point $P^{\prime}_{r}$, and can be perceived by the viewer as a spatial reconstructed point $P_{r}$. Thus it is obvious that $P^{\prime}_{o}$ and $P^{\prime}_{r}$ are conjugate points, so do $P_{o}$ and $P_{r}$, and we have $L_{o} = L_{r} = L_{c}$, $L_{{P_{o} }} = L_{{P_{r} }} = L_{P}$.

Similar to Eq. (7), the equivalent complex amplitude transmittance function of the hogel can be expressed as

10 $$t_{{{\text{Hogel}}}} (\xi ,\eta ) = T_{{{\text{Hogel}}}} (\xi ,\eta )\exp \left[ { - {\text{j}}\frac{k}{{2L_{c} }}(\xi^{2} + \eta^{2} )} \right]\fancyscript{M}[ \cdot ]$$

where the aperture function of the hogel is defined as

11 $$T_{{{\text{Hogel}}}} (\xi ,\eta ) = \left\{ {\begin{array}{*{20}c} {1,} & { \, \left| {\xi - \xi_{{O_{k} }} } \right| \le \frac{{\Delta_{H} }}{2}, \, \left| {\eta - \eta_{{O_{k} }} } \right| \le \frac{{\Delta_{H} }}{2}} \\ \, & {} \\ {0,} & { \, \left| {\xi - \xi_{{O_{k} }} } \right| > \frac{{\Delta_{H} }}{2}, \, \left| {\eta - \eta_{{O_{k} }} } \right| > \frac{{\Delta_{H} }}{2}} \\ \end{array} } \right.$$

where $\xi_{{O_{k} }}$ and $\eta_{{O_{k} }}$ are the coordinate of the point $O_{k}$, and the operator $\fancyscript{M}[ \cdot ]$ is defined as the mirror rotation operation, for any $U(\xi ,\eta )$ we have $\fancyscript{M}[U(\xi ,\eta )] = U( - \xi , - \eta )$. Thus, an upright mirrored real image with magnification $M = 1$ can always be obtained.

It should be noted that, the above equivalent model only works for the points on the central depth planes, rather than the points away from the central depth planes. However, according to the nature of holographic stereogram, this model is workable since the hogel is exposed by the effectively synthetic perspective image located on the central depth plane of the original object (plane $(x,y)$), and effectively synthetic perspective image is reconstructed on the central depth plane of the reconstructed object (plane $(x^{\prime},y^{\prime})$). Because each object point can be recorded holographically in several hogels, and then can be reconstructed directionally, thus a mirrored 3D image of the original object would be perceived by the viewer. According to Eq. (10), the characteristic of the diffraction based reconstruction is influenced by the aperture function of the hogel, thus, the imaging of the hogel based holographic stereogram can be considered as a diffraction-limited imaging system.

**Figure 4:**
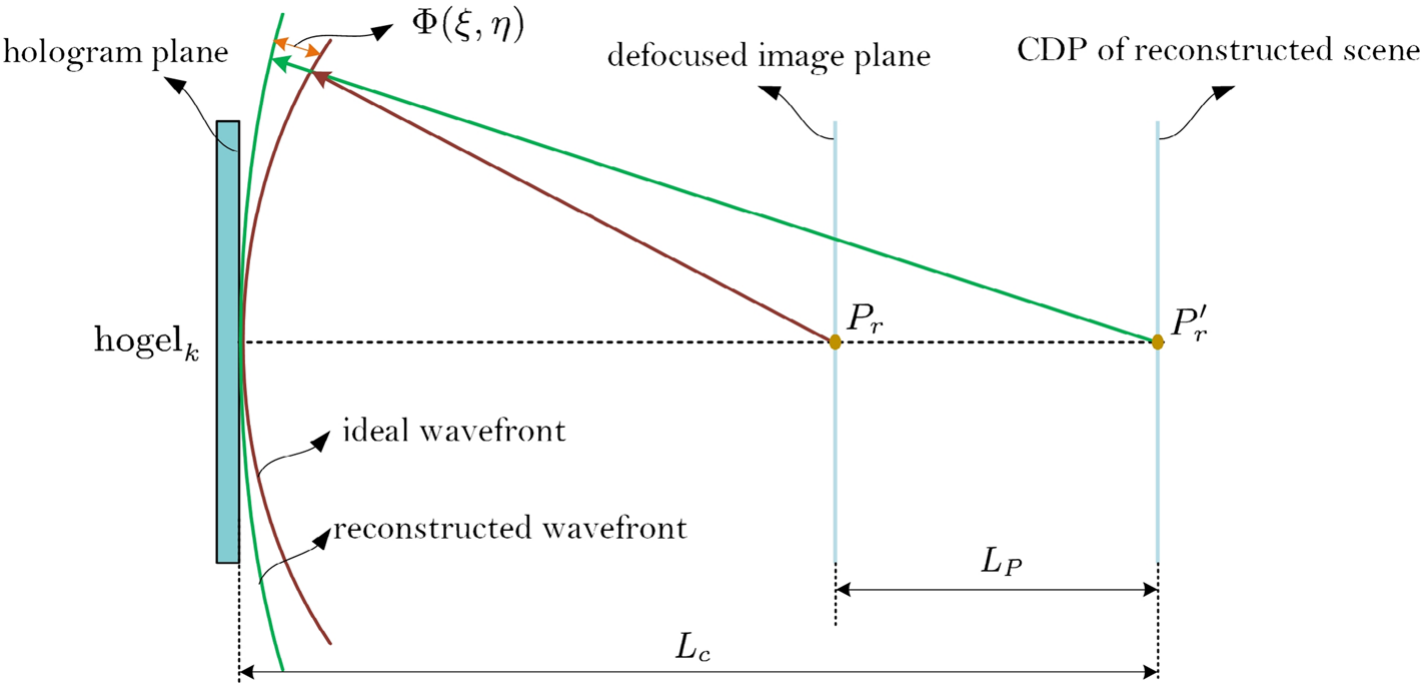
The defocused aberration for the points located away from the CDP.

Furthermore, as shown in Fig. 3b, if the all the object points locate on the central depth plane, our model of Eq. (10) is exactly true, and the imaging can be considered as a diffraction-limited imaging without aberration on the occasion of plane object which is paralleled to the hologram plane and is coincide with the central depth plane. Actually, there would often be many object points located away from the central depth plane, then the defocus aberration must be introduced and the imaging system should be considered as a diffraction-limited imaging system with aberration. As shown in Fig. 4, for ${\text{hogel}}_{k}$, it can reconstruct point $P^{\prime}_{r}$ to interpret the spatial reconstructed point $P_{r}$ with the association of other hogels. The ideal spherical wave from point $P_{r}$ propagates to the center of ${\text{hogel}}_{k}$, and then the curvature radius of spherical wave is $L_{c} - L_{P}$. However, ${\text{hogel}}_{k}$ records the wavefront from point $P^{\prime}_{r}$ located at CDP, and curvature radius of point $P^{\prime}_{r}$ is $L_{c}$. The CDP of reconstructed scene is Gauss image plane and the plane of point $P_{r}$ is defocused image plane, and the phase deviation of spherical wave between object point $P_{r}$ and image point $P^{\prime}_{r}$ is regarded as the defocused aberration. Introducing the wavefront aberration resulted from this noncoincidence, and the wave phase deviation between ideal wavefront and reconstructed wavefront is recorded as $\Phi (\xi ,\eta )$, the equivalent complex amplitude transmittance function of the hogel can be modified as

12 $$t_{{{\text{Hogel}}}} (\xi ,\eta ) = T_{{{\text{Hogel}}}} (\xi ,\eta )\exp \left[ { - {\text{j}}\frac{k}{{2L_{c} }}(\xi^{2} + \eta^{2} )} \right]\exp \left[ {{\text{j}}k\Phi (\xi ,\eta )} \right]\fancyscript{M}[ \cdot ]$$

where the wavefront aberration $\exp \left[ {{\text{j}}k\Phi (\xi ,\eta )} \right]$ equals to the phase difference between the two spherical waves

13 $$\begin{aligned} \exp \left[ {{\text{j}}k\Phi (\xi ,\eta )} \right] &= \exp \left\{ { - {\text{j}}\frac{k}{{2L_{c} }}(\xi^{2} + \eta^{2} ) - \left[ { - {\text{j}}\frac{k}{{2(L_{c} - L_{P} )}}(\xi^{2} + \eta^{2} )} \right]} \right\} \hfill \\& = \exp \left[ {{\text{j}}\frac{{k\epsilon}}{2}(\xi^{2} + \eta^{2} )} \right] \hfill \\ \end{aligned}$$

where $\epsilon$ is used to denote the defocus metric

14 $$\epsilon = \left\{ {\begin{array}{*{20}l} {\frac{1}{{(L_{c} - L_{P} )}} - \frac{1}{{L_{c} }},} \hfill & {{\text{at}}\;{\text{nearer}}\;{\text{side}}} \hfill \\ {} \hfill & {} \hfill \\ {\frac{1}{{(L_{c} + L_{P} )}} - \frac{1}{{L_{c} }},} \hfill & {{\text{at}}\;{\text{further}}\;{\text{side}}} \hfill \\ \end{array} } \right.$$

here $L_{p}$ is the absolute value of distance between the reconstructed point and the CDP which is always positive.

Thus, Eq. (12) can be rewritten as

15 $$t_{{{\text{Hogel}}}} (\xi ,\eta ) = \Gamma_{{{\text{Hogel}}}} (\xi ,\eta )\exp \left[ { - {\text{j}}\frac{k}{{2L_{c} }}(\xi^{2} + \eta^{2} )} \right]\fancyscript{M}[ \cdot ]$$

where $\Gamma (\xi ,\eta )$ denotes the generalized pupil function of the hogel

16 $$\Gamma_{{{\text{Hogel}}}} (\xi ,\eta ) = T_{{{\text{Hogel}}}} (\xi ,\eta )\exp \left[ {{\text{j}}\frac{{k\epsilon}}{2}(\xi^{2} + \eta^{2} )} \right]$$

The point spread function (PSF) for the imaging system then can be formulated as the Fourier transformation of the generalized pupil function.

17 $$h_{{{\text{Hogel}}(x^{\prime},y^{\prime})}} = {\mathcal{F}}\left\{ {\Gamma_{{{\text{Hogel}}}} (\xi ,\eta )} \right\}_{{f_{\xi } = \frac{{x^{\prime}}}{{\lambda L_{c} }},f_{\eta } = \frac{{y^{\prime}}}{{\lambda L_{c} }}}}$$

which is also the coherent impulse response of the imaging system.

## Analysis and optimization of imaging characteristics related to hogel size

**Figure 5:**
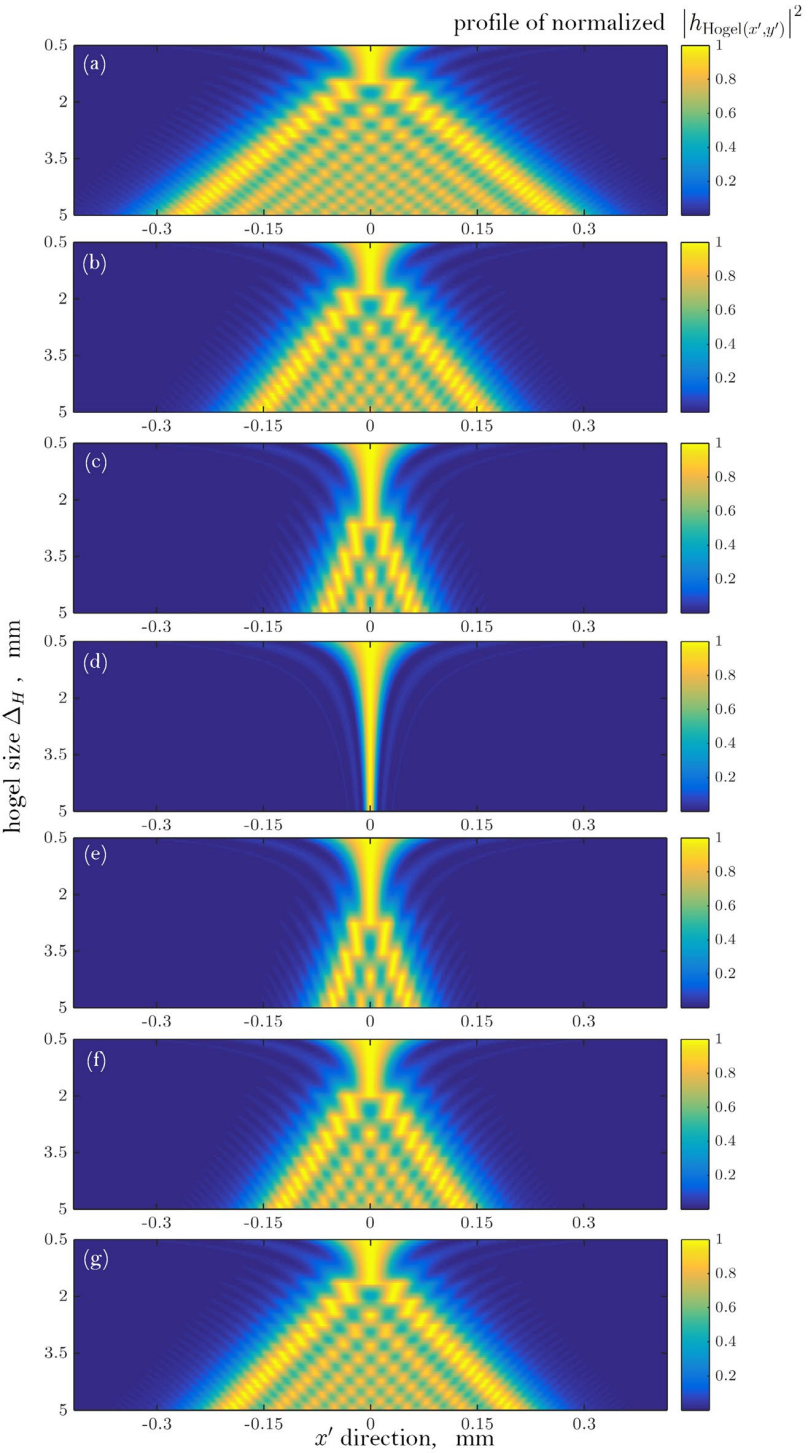
The normalized intensity of $\left| {h_{{{\text{Hogel}}(x^{\prime},y^{\prime})}} } \right|^{2}$ varying with hogel size $\Delta_{H}$ under different position offset. (**a**) $L_{p} = - 15$ mm, (**b**) $L_{p} = - 10$ mm, (**c**) $L_{p} = - 5$ mm, (**d**) $L_{p} = 0$ mm, (**e**) $L_{p} = 5$ mm, (**f**) $L_{p} = 10$ mm, (**g**) $L_{p} = 15$ mm.

Take a constructed spatial point at the nearer side of the CDP for the reconstructed scene as an example, and let $L_{c} = 12.5$ cm. Figure 5a–g show the normalized intensity of $\left| {h_{{{\text{Hogel}}(x^{\prime},y^{\prime})}} } \right|^{2}$ varying with hogel size $\Delta_{H}$ under different position offset $L_{p} = 0$ mm, ± 5 mm, ± 10 mm and ± 15 mm. It should be addressed that here the unified notation of parameter $L_{p}$ is used which is a bit different from the definition of $L_{p}$ in Eq. (14), and the positive value of $L_{p}$ means the object point locates at the farther side of CDP (see point $Q$ in Fig. 1) while the negative one stands for the nearer ones of CDP (see point $P$ in Fig. 1).

Since the PSF exhibits as a dispersion spot in most cases, especially for the object points located far away from the CDP under large hogel size, considering the special energy distribution characteristics of the reconstructed image points, the radius of the dispersion spot can no longer be measured by the traditional full width half maximum (FWHM). Here the size of the dispersion spot is defined using the method of power in the bucket (PIB)^51^,

18 $$r_{{{\text{eff}}}} \to : \, \frac{{\iint\limits_{{\sqrt {x^{{\prime}{2}} + y^{{\prime}{2}} } \le r_{{{\text{eff}}}} }} {\left| {h_{{{\text{Hogel}}}} (x^{\prime},y^{\prime})} \right|^{2} {\text{d}}x^{\prime}{\text{d}}y^{\prime}}}}{{\iint\limits_{{x^{\prime},y^{\prime} \to \infty }} {\left| {h_{{{\text{Hogel}}(x^{\prime},y^{\prime})}} } \right|^{2} {\text{d}}x^{\prime}{\text{d}}y^{\prime}}}} = \frac{1}{{\text{e}}}$$

where the number ${1 \mathord{\left/ {\vphantom {1 {\text{e}}}} \right. \kern-\nulldelimiterspace} {\text{e}}}$ is the reciprocal of the base of the natural logarithm.

**Figure 6:**
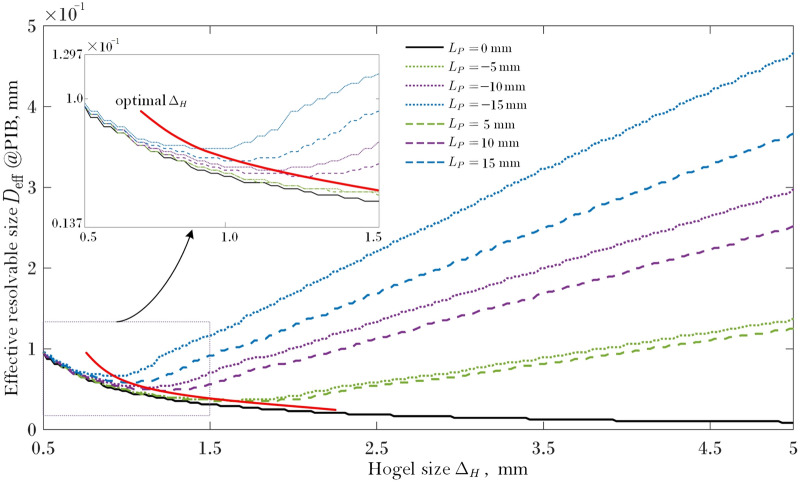
The theoretically effective resolvable size $D_{{{\text{eff}}}}$ of the reconstructed image points at different $L_{p}$ and $\Delta_{H}$.

Thus, the effective resolvable size can be defined as $D_{{{\text{eff}}}} = 2r_{{{\text{eff}}}}$. The result of theoretically effective resolvable size of the reconstructed image points at different $L_{p}$ and $\Delta_{H}$ is visually presented in Fig. 6.

As shown in Fig. 5, due to the influence of diffraction, the intensity of the point spread function $\left| {h_{{{\text{Hogel}}(x^{\prime},y^{\prime})}} } \right|^{2}$ shows a highly dispersion characteristic, especially when $L_{p}$ is not equal to zero. In addition, the effective resolvable size is defined using the method of PIB of $\left| {h_{{{\text{Hogel}}(x^{\prime},y^{\prime})}} } \right|^{2}$, thus the plots shown in Fig. 6 are not smooth enough.

When the distance between the object point and the CDP equals to 0 mm, i.e., the object point locates on the CDP, the effective resolvable size $D_{{{\text{eff}}}}$ increases with the reduced hogel size. This is due to the diffraction effect which is inversely proportional to the hogel size $\Delta_{H}$. The smaller the hogel is, the more serious the diffraction effect acts, which can result in a larger $D_{{{\text{eff}}}}$.

For the object point located out of the CDP, i.e., $L_{p} \ne 0$ mm, under the same $\Delta_{H}$, the farther the object points located away from the CDP (either on the farther side or on the nearer side), the larger the effective resolvable size is. This is because that the wavefront aberration $\exp \left[ {{\text{j}}k\Phi (\xi ,\eta )} \right]$ of the object point plays a more important role on the diffraction effect when $L_{p} \ne 0$ mm. As shown in Fig. 6, it is obvious that for the same $\Delta_{H}$, a larger $\left| {L_{p} } \right|$ relates to a more dispersed image spot along with a larger $D_{{{\text{eff}}}}$. Even for the two object points with the same $\left| {L_{p} } \right|$ which means they are mutually mirrored by CDP, there is a difference between the $D_{{{\text{eff}}}}$ of the object point on the farther side and that on the nearer side. It is shown that $D_{{{\text{eff}}}}$ for the object point on the farther side is a little lower than that of the points on the nearer side. This is due to the defocus metric $\epsilon$ is different for the two mirrored object point. When the $\left| {L_{p} } \right|$ is small enough ($\left| {L_{P} \ll L_{c} } \right|$), we have

$$\epsilon = \left\{ {\begin{array}{*{20}l} {\frac{1}{{(L_{c} - \left| {L_{P} } \right|)}} - \frac{1}{{L_{c} }} \approx \frac{{\left| {L_{P} } \right|}}{{L_{c}^{2} }} \approx 0,} \hfill & {{\text{at}}\;{\text{nearer}}\;{\text{side}}} \hfill \\ {} \hfill & {} \hfill \\ {\frac{1}{{(L_{c} + \left| {L_{P} } \right|)}} - \frac{1}{{L_{c} }} \approx - \frac{{\left| {L_{P} } \right|}}{{L_{c}^{2} }} \approx 0,} \hfill & {{\text{at}}\;{\text{further}}\;{\text{side}}} \hfill \\ \end{array} } \right.$$

which shows that their $\epsilon$ have the same amplitude, i.e., $\left| {\epsilon_{{{\text{nearer}}}} /\epsilon_{{{\text{further}}}} } \right| \approx 1$, and both tend to zero, thus the difference between their $D_{{{\text{eff}}}}$ is also very small (see Fig. 6). However, if $\left| {L_{p} } \right|$ is large enough, the $\epsilon$ then can be expressed as

$$\epsilon = \left\{ {\begin{array}{*{20}l} {\frac{1}{{(L_{c} - \left| {L_{P} } \right|)}} - \frac{1}{{L_{c} }} = \frac{{\left| {L_{P} } \right|}}{{L_{c} (L_{c} - \left| {L_{P} } \right|)}},} \hfill & {{\text{at}}\;{\text{nearer}}\;{\text{side}}} \hfill \\ \, \hfill & {} \hfill \\ {\frac{1}{{(L_{c} + \left| {L_{P} } \right|)}} - \frac{1}{{L_{c} }} = - \frac{{\left| {L_{P} } \right|}}{{L_{c} (L_{c} + \left| {L_{P} } \right|)}},} \hfill & {{\text{at}}\;{\text{further}}\;{\text{side}}} \hfill \\ \end{array} } \right.$$

which results in $\left| {\epsilon_{{{\text{nearer}}}} /\epsilon_{{{\text{further}}}} } \right| = \, \left| {(L_{c} + \left| {L_{P} } \right|)/(L_{c} - \left| {L_{P} } \right|)} \right| > 1$. It can be seen that when $\left| {L_{P} } \right|$ is large enough, the nearer object point has a much larger defocus metric than that its mirrored point at the further side, therefore, the difference of $D_{{{\text{eff}}}}$ between these two mirrored object points becomes markable, and the larger the $\left| {L_{P} } \right|$, the larger this difference is, which can be seen obviously in Fig. 6. For example, the difference of $D_{{{\text{eff}}}}$ at $\left| {L_{P} } \right|$= 15 mm is much more distinct than that at $\left| {L_{P} } \right|$= 5 mm.

In addition, as shown in Fig. 6, under a given $L_{P}$ ($L_{p} \ne 0$), an optimal hogel size $\Delta_{H}$ can be achieved with which the $D_{{{\text{eff}}}}$ is minimized, and this relates to the highest viewing resolution for object points located at the given $L_{P}$. For aberrationless plane $L_{p} = 0$, the optimal hogel size $\Delta_{H}$ trends to infinity since the imaging will be ideal when the aperture size is infinity along with the absence of aberration. This is because that the imaging performance of the diffraction limited system, especially the resolution, is dominated by the composite effects of the size of the aperture ($\Delta_{H}$) and the defocused distance ($L_{P}$), so does the holographic stereogram. In hogel based holographic stereogram, the $D_{{{\text{eff}}}}$ is decreased along with the reduction of the $\Delta_{H}$, but is increased as the $L_{P}$ is increased. Under a small $\Delta_{H}$, the diffraction effect plays a dominant part on $D_{{{\text{eff}}}}$. However, when the $L_{P}$ is large enough, the influence of the defocused aberration then also plays an important role. Thus, an optimal hogel size is existed under a given $L_{P}$ ($L_{p} \ne 0$), which corresponds to a minimized resolvable spot size $D_{{{\text{eff}}}}$. From Fig. 6, it can be observed that the optimal hogel size $\Delta_{H}$ is increased with the decreasing of $\left| {L_{P} } \right|$, and when $\left| {L_{P} } \right|$ tends to 0, the optimal $\Delta_{H}$ tends to infinity, which means that if the defocused aberration is toward to 0, an infinity aperture is the optimal hogel which let both all the low and high spatial frequencies pass through, and can result in an ideal geometrical imaging, i.e., an object point can be imaged as an ideal image point, thus $D_{{{\text{eff}}}} \to 0$.

From the curve of $L_{P} =$ 15 mm or $L_{P} = -$ 15 mm, it can be inferred that the resolution of the reconstructed image points sharply deteriorates with the increase of the size of hogel. Comparing minimum values of curves with different $L_{P}$ in Fig. 6, it can be found that the optimal size of hogel decreases with the increase of the absolute value of $L_{P}$. That is to say, in the case of large aberration, it is necessary to use a smaller hogel size to achieve a higher quality reconstruction image. When the location of CDP ($L_{c}$) and the defocused distance ($L_{P}$) are certain, the optimum hogel size can be calculated from Eq. (10) to Eq. (18). However, above model of diffraction-limited imaging is not applicable to all types of holographic stereogram, for example, the CHIMER printer system proposed by Yves Gentet et al*.*^52^, the optimal hogel size cannot be obtained correctly due to different optical setup of the printing system.

**Figure 7:**
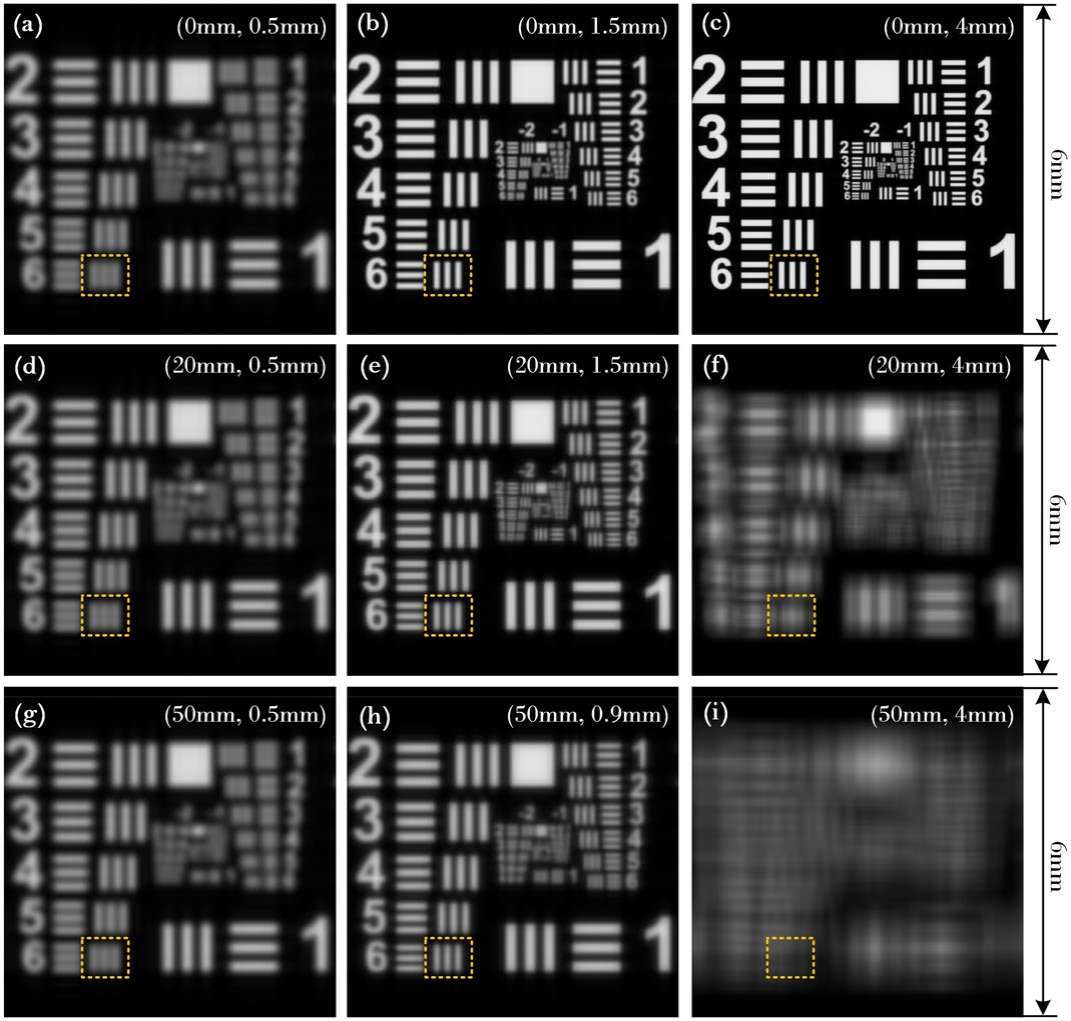
Degradation of the imaging quality for the reconstructed images under different positions of image plane $L_{p}$ with different hogels sizes $\Delta_{H}$, (**a**) $L_{p} =$ 0, $\Delta_{H} =$ 0.5 mm; (**b**) $L_{p} =$ 0, $\Delta_{H} =$ 1.5 mm; (**c**) $L_{p} =$ 0, $\Delta_{H} =$ 4 mm; (**d**) $L_{p} =$ 20 mm, $\Delta_{H} =$ 0.5 mm; (**e**) $L_{p} =$ 20 mm, $\Delta_{H} =$ 1.5 mm; (**f**) $L_{p} =$ 20 mm, $\Delta_{H} =$ 4 mm; (**g**) $L_{p} =$ 50 mm, $\Delta_{H} =$ 0.5 mm; (**h**) $L_{p} =$ 50 mm, $\Delta_{H} =$ 0.9 mm; (**i**) $L_{p} =$ 50 mm, $\Delta_{H} =$ 4 mm.

The resolution board of USAF1951 is applied as the object to perform the simulated verification, and the theoretical reconstructed results are shown in Fig. 7. Figure 7a–c show the reconstructed images when the object is located at CDP ($L_{P} = 0$), and it is obvious that the viewing resolution is increasing along with the enlargement of hogel size. When $L_{P} = 5$ mm (see Fig. 7d–f), the sharpest reconstruction is achieved at the optimal hogel size $\Delta_{H} = 1.5$ mm, and the resolution of the reconstruction is deteriorated when the object is placed off away from the CDP. The similar results are also demonstrated at $L_{P} = 15$ mm under different hogel sizes, however, the optimal hogel size is shifted to $\Delta_{H} = 0.9$ mm. The resolution deterioration is also occurred along with the increasing of the position offset $L_{P}$ when the hogel size $\Delta_{H}$ is fixed, which can be seen from Fig. 7c, f, i. Since the resolution of the reconstruction is inversely proportional to that of the effective resolvable size $D_{{{\text{eff}}}}$, thus, these theoretical simulations are well agreed with the modelled ones.

## Experimental results and discussions

**Figure 8:**
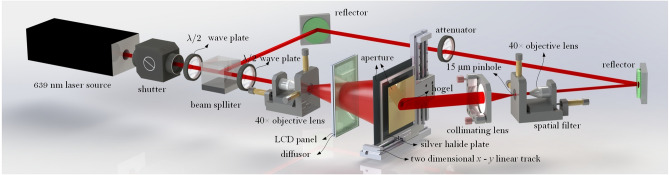
Experiment setup of the printing of full-parallax EPISM based holographic stereogram.

The holographic stereogram printing system is illustrated in Fig. 8. A continuous wave 400 mW 639 nm single-longitudinal-mode linearly polarized solid-state laser (model CNIMSL-FN-639 @CNI) is utilized as the laser source. A mechatronic shutter (model SSH-C2B @Sigma Koki) modulates the laser output to control the exposure time of the holographic plate. The modulated laser is splitted into the object arm and the reference arm by a $\lambda /2$ wave plate and a polarization-dependent beam splitter. The power ratio between the object beam and the reference beam can be adjusted conveniently by rotating the $\lambda /2$ wave plate before the beam splitter. Another $\lambda /2$ wave plate is placed in the object arm, which is aimed to adjust the polarization direction of the object beam as the same as that of the reference beam. The 40× objective lens is used to expand the object beam to illuminate the effective area of the LCD, and the effectively synthetic perspective images are loaded and projected on the adapted LCD panel (model VVX09F035M20 @Panasonic) to expose the holographic plate. The LCD is 8.9 inches, with 1920 × 1,200 pixels and 0.1 mm pixel spacing, whose backlight module is removed except the diffusor. After passing through the LCD, the object beam is projected onto the silver halide plate which is placed 15 cm away from the LCD panel and the light intensity is $\Delta E$ = 1,250 μJ/cm^2^@639 nm. Two square apertures are positioned before and after the silver halide plate to block the unexpected light and form the hogels. The different size hogel can be printed by changing the different apertures such as 2 mm, 4 mm and 10 mm. The power of the reference beam can be changed by a attenuator placed between two reflectors, and then the power ratio of object/reference can be further adjusted. A spatial filter comprised of a 40× objective and a 15 μm pinhole is used to filter out the higher spatial frequency and a collimating lens with $f = 150$ mm is placed behind the spatial filter, which is used to collimate the reference beam as planar wave. The pinhole is place on the common focus point of both the objective and the collimated lens. The angle between the reference beam and the object beam is about 30º. A two dimensional $x - y$ linear track (model KSA300@Zolix) is used to move the silver halide plate to the position of the next hogel after the exposure of the former one is finished. A synchronous control system is developed to synchronize the shutter, the LCD, and linear track.

**Figure 9:**
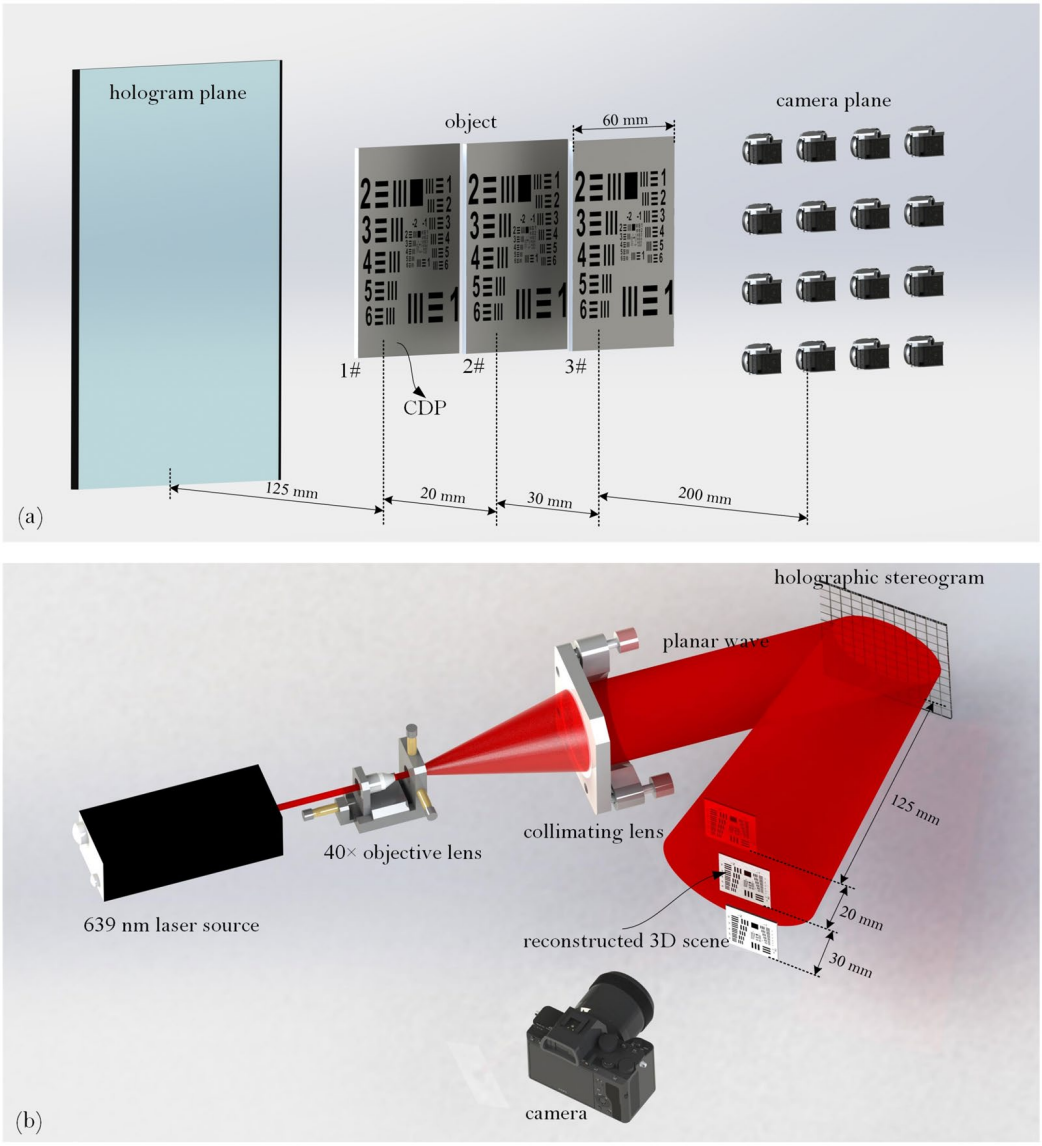
(**a**) Geometrical configuration of the 3D object sampling, and (**b**) the setup of the reconstructing.

An additional optical experiment was also carried out to verified the theoretical prediction. The geometrical relationship is shown in Fig. 9a. The CDP is set as 12.5 cm away from the hologram. The object consisted of three resolution boards of USAF1951 (1#, 2# and 3#) is used as the target object, and is positioned at 12.5, 14.5 and 17.5 cm away from the hologram plane which yields to $L_{P} =$ 0, 20, and 50 mm, respectively. Under each given $L_{P}$, three different hogels sizes, $\Delta_{H} =$ 2, 4 and 10 mm are also employed. The holographic stereogram is fabricated based on the EPISM method proposed by our group^39^. 139 × 139 perspective images are obtained with the resolution of each 1,000 × 1,000. These perspective images are resynthesized to generate the effectively synthetic perspective image based on EPISM. The number of effectively synthetic perspective image is 30 × 30, 15 × 15 and 6 × 6 respectively, and the resolution of which is 1,000 × 1,000.

**Figure 10:**
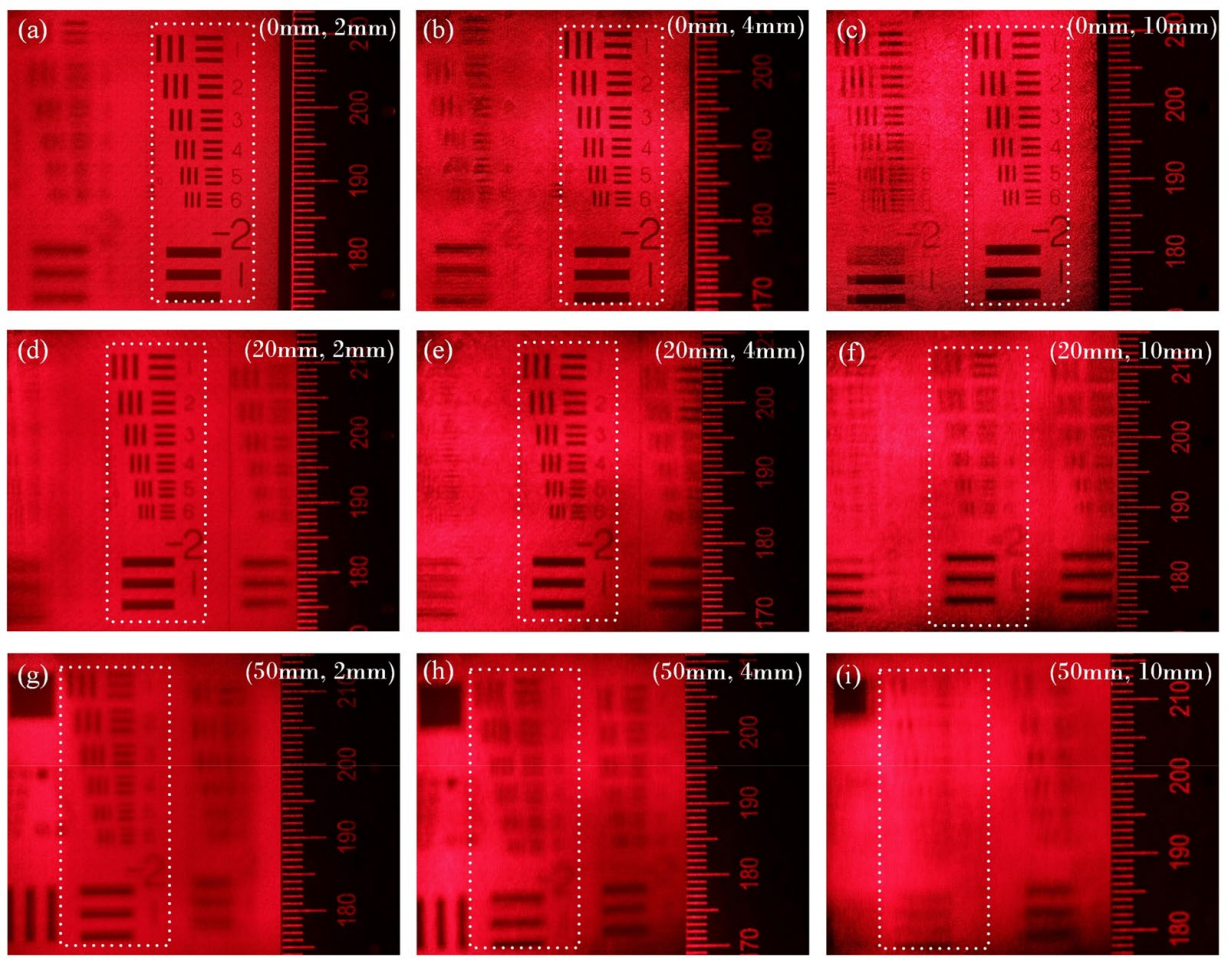
The images of optical reconstruction of the resolution board under different positions of image plane $L_{P}$ with different hogels sizes $\Delta_{H}$, (**a**) $L_{P} =$ 0, $\Delta_{H} =$ 2 mm; (**b**) $L_{P} =$ 0, $\Delta_{H} =$ 4 mm; (**c**) $L_{P} =$ 0, $\Delta_{H} =$ 10 mm; (**d**) $L_{P} =$ 20 mm, $\Delta_{H} =$ 2 mm; (**e**) $L_{P} =$ 20 mm, $\Delta_{H} =$ 4 mm; (**f**) $L_{P} =$ 20 mm, $\Delta_{H} =$ 10 mm; (**g**) $L_{P} =$ 50 mm, $\Delta_{H} =$ 2 mm; (**h**) $L_{P} =$ 50 mm, $\Delta_{H} =$ 4 mm; (**i**) $L_{P} =$ 50 mm, $\Delta_{H} =$ 10 mm.

Experiment setup of the reconstruction of holographic stereogram is shown in Fig. 9b. A 40× objective lens is used to expand the laser beam and a collimating lens with $f = 150$ mm is used to collimate the beam as planar wave to illuminate the holographic stereogram. The incident angle of illuminating beam is consistent with the incident angle of reference beam. Canon5D Mark III is used to capture the reconstructed images, which is equipped with Canon RF 70–200 mm f/2.8L IS USM Lens. In order to evaluate the view-flipping effect of the reconstructed image, the focal length of the lens is fixed to 100 mm, the aperture setting is 5.6, which leads to an actual aperture size of about 17.86 mm. Compared with the diameter of the pupil of the human eye (~ 4 mm), the image taken by this camera is equivalent to the superposition of images, which is perceived by the viewer when he/she moves 13.86 mm laterally from 35 cm away (parallel to the hologram plane). Thus, the level of the view-flipping effect can be reflected on the severity of ghosting in the picture. The results are shown in Fig. 10a–i. The reconstructed images are captured by a camera which focuses on the three different resolution boards respectively, while a rule is also placed on its focused plane as a reference. It can be seen that under the same $\Delta_{H}$, the reconstructed 3D scene has a remarkable deterioration and blur on $L_{P} =$ 20 mm and 50 mm, especially that of the latter is much worse than the former one, compared to the clear and sharp reconstruction at $L_{P} =$ 0 mm. Comparing the reconstruction at the same $L_{P}$ under different $\Delta_{H}$, we can find that the imaging quality of $\Delta_{H} = 2$ mm is better than $\Delta_{H} = 4$ mm while that of $\Delta_{H} = 4$ mm is better than $\Delta_{H} = 10$ mm, and the larger the object deviates from the CDP, the more obvious optimization of the size of hogel to the imaging quality appears. The experimental results also indicate that there is none view-flipping effect existed on the reconstruction of the 1# resolution board whose $L_{P} =$ 0, whatever the hogel size $\Delta_{H}$ is. For resolution board 2# or 3#, the smaller the hogel size $\Delta_{H}$ is, the slighter the view-flipping is behaving. On the other hand, if we fixed the hogel size $\Delta_{H}$, the view-flipping effect reduces along with the decreasing of the distance from the resolution board to the CDP. It indicates that the experimental results corroborate the theoretical prediction quit well.

**Figure 11:**
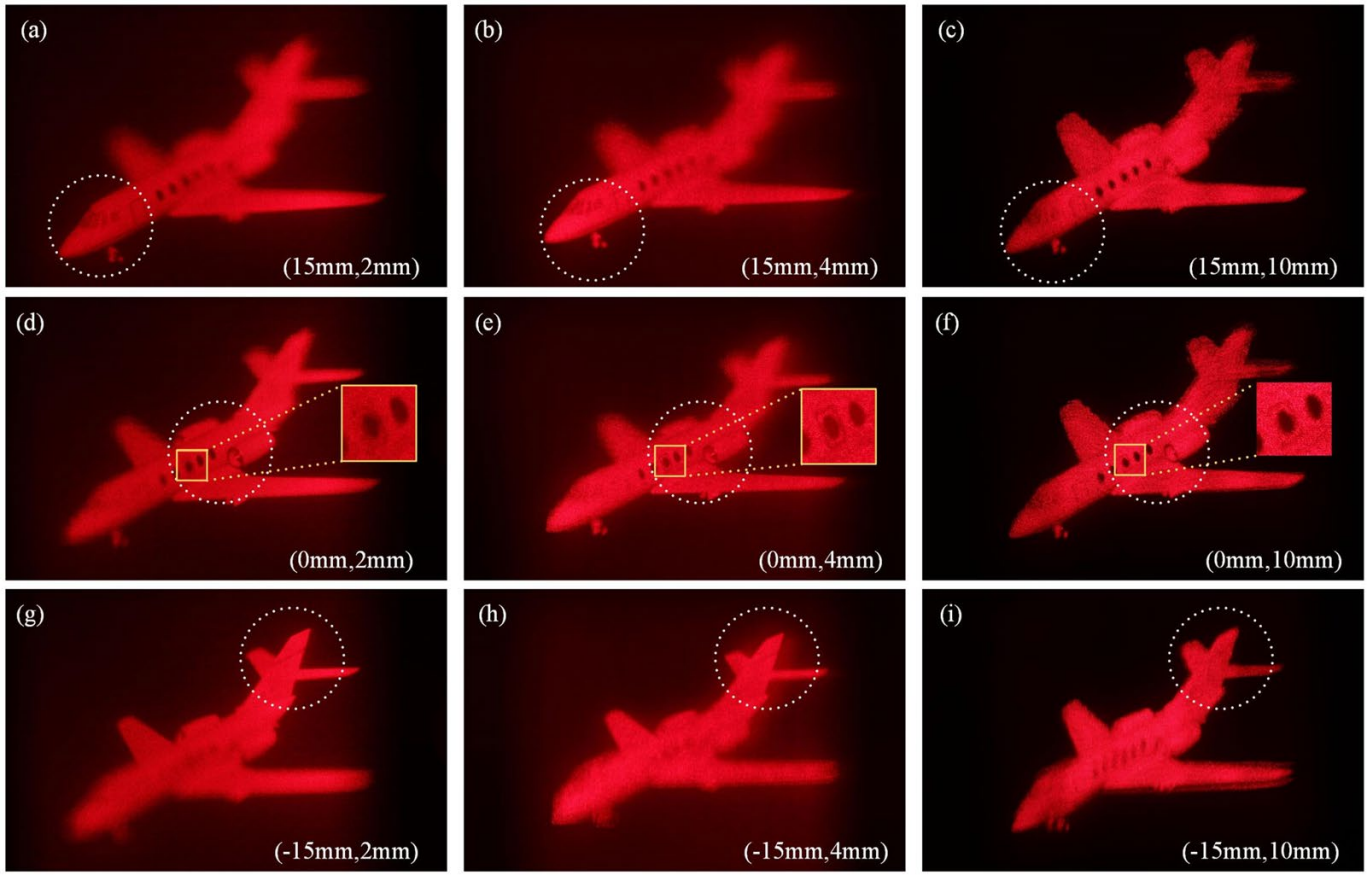
The images of optical reconstruction of the 3D aircraft under different positions of image plane $L_{P}$ with different hogel sizes $\Delta_{H}$, (**a**) $L_{P} =$ 15 mm, $\Delta_{H} =$ 2 mm; (**b**) $L_{P} =$ 15 mm, $\Delta_{H} =$ 4 mm; (**c**) $L_{P} =$ 15 mm, $\Delta_{H} =$ 10 mm; (**d**) $L_{P} =$ 0, $\Delta_{H} =$ 2 mm; (**e**) $L_{P} =$ 0, $\Delta_{H} =$ 4 mm; (**f**) $L_{P} =$ 0, $\Delta_{H} =$ 10 mm; (**g**) $L_{P} =-$ 15 mm, $\Delta_{H} =$ 2 mm; (**h**) $L_{P} =-$ 15 mm, $\Delta_{H} =$ 4 mm; (**i**) $L_{P} =-$ 15 mm, $\Delta_{H} =$ 10 mm.

Furthermore, a three-dimensional aircraft model presented in Fig. 1 is used as a relatively complicated 3D scene to verify the validity of the theoretical analysis. The longitudinal depth of the inclined aircraft model, $\Delta_{S}$, is about 30 mm, and the CDP is placed at the center of the aircraft and is positioned 125 mm away from hologram plane. Figure 11a, d, g show the reconstructed images focused on the head, body and tail part of the aircraft respectively when the size of the hogel $\Delta_{H}$ is 2 mm. As we change $\Delta_{H}$ to 4 mm and 10 mm, the reconstructed images are shown in Fig. 11b, e, h and c, f, i.

From Fig. 6, it can be seen that the optimal size of the hogel size is about 1.2 mm. For the head and tail part of the aircraft, comparing the reconstructed images under different hogel size $\Delta_{H}$, it can be observed that the reconstructed image quality will deteriorate with the increase of the size of the hogel, which is in accordance with the above theoretical analysis. For the body part of the aircraft model, i.e. the points near or on the CDP, their reconstructed images have a similar resolution for the three different selected hogel size, as shown in Fig. 11a, d, g. This is also in agreement with theoretical prediction. According to Fig. 6, it can be inferred that the resolution of the reconstructed image of the points near or on the CDP ($L_{P} =$ 0, 5 and − 5 mm) decreases slightly as the hogel size gets smaller, which coincides with the experimental results. Meanwhile, for $\Delta_{H} =$ 2 mm, the nearly smooth motion parallax can both be obtained when observing from different viewing angles, even for the head and tail part of the aircraft which is located far away from the CDP. However, the motion parallax is not continuous any longer for the head and tail part of the aircraft when $\Delta_{H} =$ 10 mm, and the obvious view-flipping effect can be observed, but the partial body of the aircraft straddled the CDP with about $\left| {L_{P} } \right| < 5$ mm is almost free of view-flipping effect, which corroborate the previous analysis. The view-flipping effect for the head and tail part of the aircraft is stronger than that of the body, and the viewing resolution is relatively lower. That is, the quality of the reconstructed image for the head and tail part is worse than that of the body. When the hogel size $\Delta_{H}$ changes from 10 to 4 mm and then to 2 mm, the view-flipping effect gradually weakens, and the whole viewing resolution enhances because smaller hogel size improves the effective resolvable size caused by Gaussian defocus aberration to some extent. However, it should also be pointed out that the minimization of both the view-flipping effect and the reconstruction spatial resolution cannot be achieved at the same time. There is a somehow tradeoff between these two visual performance, and the main achievement of this work is to realize an acceptable compromise between the view-flipping effect and the whole viewing reconstruction spatial resolution for the given 3D scene by optimizing the hogel’s size. In addition, comparing unfocused aircraft tail shown in Fig. 11a–c, the virtualization and blurring effect should be observed, and this virtualization and blurring effect should get severely along with the increasing of the $\Delta_{H}$. According to experimental results, however, this is true for Fig. 11a, b, but an abnormal result is obtained in Fig. 11c. Specifically, obvious aliasing artifacts can be seen in Fig. 11c, which is attributed to the insufficient number of perspective images used to generate the synthetical effective perspective images when the hogel size $\Delta_{H}$ is 10 mm. It should be noted that, in our experiment, limited by the low power of laser and the experimental conditions, it is difficult to make a hologram with the hogel size less than 2 mm. Thus, the experimental results did not serve a direct verification of the optimal hogel size shown in Fig. 6. However, noticed that the value of the optimal hogel size is related to a certain depth, the exist of the optimal hogel size can still be proved alternatively by the experiment. As depicted in the inset images of Fig. 11d–f, there is a central window surrounded by a black line frame which is close to the CDP. It is obvious that the clarity and sharpness of the inset image of Fig. 11e is higher than that of Fig. 11d, f, especially for the detailed line frame as well as the edge of the central window. This implies that, for this certain depth, there is an optimal hogel size existed between 2 and 10 mm, or around 4 mm. When the hogel size is smaller than the optimal one, such as 2 mm, the diffraction effect of limited aperture will reduce the viewing resolution of the reconstruction. On contrast, the increase of wavefront aberration begins to play a dominant role when the hogel size is larger than the optimal one, such as 10 mm, which will also cause the reduction of viewing resolution.

## Conclusion

In summary, we analyze the view-flipping effect of the hogel based holographic stereogram, and establish the diffraction-limited imaging model to optimize the reconstruction resolution. Theoretical analysis shows that the hogel size should be diminished if the view-flipping effect is to reduce or eliminate. However, the diffraction effect resulted from the small window of hogel must be taken into account. Then the defocus aberration is introduced and a model of diffraction-limited imaging system for the hogel based holographic stereogram is established, and the point spread function for the imaging system is obtained from the generalized pupil function of the hogel. The effective resolvable size is used to evaluate the diffraction effect and further the reconstruction resolution. The theoretical results predict that there is an optimal hogel size existed for the certain depth of scene. The theoretical and experimental results indicate that the modeling of modulation characteristics of hogel based holographic stereogram agrees well with the experiments, and our method will be helpful to improve the imaging quality of holographic stereogram.
